# Multidisciplinary Simulation for Blunt and Penetrating Pediatric Trauma Utilizing Standard and Rapid Cycle Deliberate Practice Models

**DOI:** 10.15766/mep_2374-8265.11390

**Published:** 2024-03-19

**Authors:** Erin B. Henkel, Daniel Lemke, Daniel Rubalcava, Bindi Naik-Mathuria, Katherine M. Gautreaux, Jeannie Eggers, Cara Doughty

**Affiliations:** 1 Assistant Professor, Department of Pediatrics, Baylor College of Medicine; Associate Trauma Medical Director, Division of Pediatric Emergency Medicine, Texas Children's Hospital; 2 Associate Professor, Division of Pediatric Emergency Medicine, Department of Pediatrics, Baylor College of Medicine; Associate Medical Director of Simulation Center, Texas Children's Hospital; 3 Assistant Professor, Department of Pediatrics, Baylor College of Medicine; Associate Trauma Director, Division of Pediatric Emergency Medicine, Texas Children's Hospital; 4 Professor, Division of Pediatric Surgery, Department of Surgery, University of Texas Medical Branch; 5 Trauma Education Coordinator, Trauma Services, Texas Children's Hospital; 6 Manager for Quality Education and Simulation, Texas Children's Hospital; 7 Associate Professor, Department of Pediatrics, Baylor College of Medicine; Medical Director, Simulation, Texas Children's Hospital

**Keywords:** Trauma, Rapid Cycle Deliberate Practice, Clinical/Procedural Skills Training, Emergency Medicine, Pediatric Emergency Medicine, Pediatrics, Simulation, Surgery - General, Surgery - Pediatric

## Abstract

**Introduction:**

Pediatric trauma resuscitations are low-frequency, high-stakes events that require skilled multidisciplinary teams with strong medical knowledge and communication skills.

**Methods:**

This pediatric trauma simulation training session included two cases and formats. The first case was designed in a traditional format and featured a 12-month-old child with inflicted blunt head and abdominal trauma. The second case was organized in successive rounds utilizing the rapid cycle deliberate practice (RCDP) model and featured an 18-month-old with gunshot wounds to the abdomen and chest. Educational objectives included effective communication in a multidisciplinary team, timely completion of primary and secondary surveys, awareness of systems and processes related to trauma care, and increasing competency with low-frequency pediatric trauma skills. Necessary equipment included high-fidelity toddler-sized mannequins, chest tube task trainer or applicable mannequin and equipment, intubation equipment and supplies, intraosseous access, and blood products with rapid delivery infusers. This training session was designed for learners in a multidisciplinary team including physician trainees, nurses, and advanced practice providers; adjustments could be made to the team members as desired.

**Results:**

Quantitative and qualitative evaluations demonstrated high learner satisfaction and engagement, particularly in the RCDP style of learning.

**Discussion:**

Multidisciplinary team practice of pediatric trauma scenarios, particularly utilizing the RCDP simulation model, provides the opportunity to improve teamwork and communication, practice procedural skills, and deepen team members' understanding of and comfort with trauma resuscitations.

## Educational Objectives

By the end of this activity, learners will be able to:

1. Demonstrate effective communication as the leader of a code 1 pediatric trauma resuscitation.
2. Perform a timely primary survey of a critically injured pediatric trauma patient.
3. Identify necessary emergent interventions at each step of the primary survey.
4. Use crisis resource management skills of assigning clear roles and using closed-loop communication and shared mental models.
5. Adjust priorities of management for a pediatric patient with blunt versus penetrating traumatic injuries.

## Introduction

Trauma resuscitations are demanding and dynamic events that require an efficient interdisciplinary team directed by a strong team leader. Paramount to the success of trauma resuscitation are the rapid identification of life-threatening injuries and initiation of lifesaving interventions. Because high-stakes trauma activations may be infrequent, simulation has been shown to be effective in improving trauma team performance.^1^ Often, children who present with abusive injuries are not identified on first presentation and subsequently present with new injuries, posing a higher risk of mortality.^2,3^ Similarly, penetrating trauma is infrequent at children's hospitals, and specific training for such injuries is not emphasized in the current versions of Advanced Trauma Life Support (ATLS).^4^

Simulation can be an effective method to improve awareness, evaluation, and management of both abusive and penetrating traumatic injuries.^5^ Simulation-based training has been shown to be an effective tool in preparing for time-sensitive multidisciplinary trauma resuscitations. Simulation improves trauma team functioning^6^ and decreases time to critical trauma resuscitative procedures.^7^ Pediatric multidisciplinary trauma simulation has specifically been shown to improve provider confidence^8^ and may lead to lower risk of mortality in pediatric trauma centers.^9^ Rapid cycle deliberate practice (RCDP) is a type of simulation-based training, first described by Hunt and colleagues in 2014, in which learners “rapidly cycle between deliberate practice and directed feedback until skill mastery is achieved.”^10^ Among the positive outcomes associated with RCDP training are high learner satisfaction and increased confidence, acquisition of knowledge and skills, and decreased time to critical tasks.^11^ RCDP simulation training in pediatric trauma has demonstrated participant improvement not just in the practice setting but also in actual patient care.^12^

The pediatric trauma simulation cases previously published in *MedEdPORTAL* primarily include blunt injury mechanisms at the medical student and resident learning levels.^13–15^ The current publication offers the novel aspect of including both blunt and penetrating injury mechanisms and features key teaching points related to management strategies specific to penetrating injuries. Additionally, this session embraces both traditional and RCDP formats that can aid learners and instructors with opportunities for teaching points, learning, and solidifying best practices. The session is written to address more experienced learners—pediatric emergency medicine (PEM) fellows, surgery upper-level residents, pediatric surgery fellows, and trauma-trained nurses—but can be modified for a variety of multidisciplinary settings.

## Methods

### Development

A prior quality improvement project at our institution involving video review of code 1 trauma activations and resuscitations using the T-NOTECHS^16^ scoring tool identified a need for improved communication and team-based evaluation in high-level trauma activations. Based on that need, we assembled a team of key stakeholders, including experts in trauma, PEM, nursing, and simulation. Stakeholders met to identify learning objectives, develop case scenarios, and refine and test the scenarios. Key goals included team communication, best-practice trauma management, and system-specific education regarding trauma processes. Physician-specific skills training included chest tube placement, with a concurrent nursing-specific skill of chest tube setup with vacuum drainage system, as well as rapid infuser blood product and fluid administration and electronic medical record (EMR) documentation using a trauma narrator flow sheet.

Our multidisciplinary learner groups consisted of providers functioning on the trauma resuscitation team—PEM fellows, surgery residents, emergency center (EC) and trauma surgery advanced practice providers (APPs), emergency nurses, respiratory therapists (RTs), and emergency medical technicians (EMTs). The cases stressed essential ATLS trauma management principles, including timely completion of primary and secondary surveys, performance of critical resuscitative interventions, utilization of adjunctive studies, and appropriate and timely disposition. There was a particular emphasis on penetrating trauma, as these injuries have become increasingly more common in children's hospitals where historical experience has primarily been blunt injuries. Simulation debriefing and feedback focused on completion of tasks but also stressed components of effective communication and teamwork. While our institutional focus was on pediatric providers, emergency providers who do not routinely care for pediatric trauma patients could potentially benefit from the session as well.

### Equipment/Environment

We used a high-fidelity SimBaby Classic mannequin (Laerdal) with the patient ages being 12 and 18 months, respectively, for the blunt and penetrating trauma cases. Depending on available resources, the cases could be modified to a small or medium child mannequin with appropriate vital sign adjustments for age. Simulation center personnel assisted with mannequin moulage and setup. We used purple eye shadow for bruising moulage, prefabricated bullet wounds, diapers, and a patient gown that required removal during the primary survey. Equipment included peripheral intravenous and interosseous needles, nonrebreather masks, intubation equipment, length-based resuscitation tape, and a cervical collar of the appropriate size. Instructor guides available for reference included the cases, team management objectives and actions, ideal choreography for completion of primary and secondary surveys, and lab values and imaging results for both cases (Appendices A and B). A pediatric Glasgow Coma Scale^17^ (GCS) was posted for team use during the scenarios. We utilized a separate task trainer for needle decompression and chest tube placement for the second case, as well as an 18-gauge needle, chest tube, chest tube placement kit, and pleural drainage kit. If these supplies are not available, this skill could be omitted or discussed without real-time practice. We provided bags of normal saline, simulated blood, and a rapid infuser with tubing available for nursing practice following the simulation. A test environment was constructed in our EMR system, and a workstation was made available to nurses during simulation for practicing documentation. Both cases could be run in a low-fidelity manner based on available resources, if necessary.

### Personnel

A minimum of three instructors led each session, one each from PEM, pediatric surgery/trauma, and nursing. When available, a fourth instructor and/or simulation center staff member was useful to operate the high-fidelity mannequin and co-debrief. Instructors played the roles of emergency medical services (EMS) personnel at arrival (nursing or PEM) and attending surgeon arrival for handoff. Embedded participants could also play these roles. Most of the instructors and, at minimum, one of the physician instructors per session had completed the simulation instructor course at our institution. These instructors had specific training in both traditional and RCDP case design and debriefing, co-debriefing, and PEARLS (Promoting Excellence and Reflective Learning in Simulation) debriefing.^18^ At the time of submission, all instructors were active ATLS instructors as well. While this degree of training is not necessary to run these sessions, basic knowledge of simulation teaching including RCDP principles is recommended.

### Learner Group

We created multidisciplinary learner groups with a minimum of three members, one each from PEM, surgery, and nursing. Initially, the team included one or two of each of the following: PEM fellows, surgery residents, and EC nurses. Learner groups expanded to include EC APPs, all levels of surgery residents, pediatric surgery fellows, surgical APPs, EC EMTs, and RTs. Ideal teams included five members: one from PEM, two from surgery, and either two from nursing or one nurse and one EMT. We recommended all physician and APP learners complete ATLS prior to participating, so that primary and secondary assessments of trauma patients were assumed background knowledge. EC nurses were required to have completed the Trauma Nursing Core Course (TNCC) prior to participation.^19^ Our core trauma nursing group participated as often as possible to ensure thorough knowledge of pediatric emergency nursing and TNCC core concepts, as well as to provide perspective as active members of the trauma team.

### Implementation

The first case involved a 12-month-old boy who reportedly fell off a changing table and had a seizure (Appendix A). Goals included identifying this presentation as a traumatic head injury and potential child abuse given the exam findings. Setup included moulage bruising on the lower abdomen beneath the diaper, requiring a full exposure by the team to uncover concern for blunt abdominal trauma. The team assembled outside the room and/or away from the patient, where an EMS prearrival report was given to allow the team leader to assemble and organize the team prior to patient arrival. After 1–2 minutes for team organization, an instructor indicated the patient had arrived and gave an EMS report that included declining mental status and drowsiness en route. The team leader directed a primary evaluation, including findings of borderline hypotension (76/30) and altered mental status (GCS 9; instructors gave individual components when asked). Full exposure revealed bruising and concern for blunt abdominal trauma. The team lead directed resuscitation, including bolus of fluids versus blood products, intubation for airway protection given the rapid decline in mental status from GCS 15 to 9, code 1 trauma activation, neurosurgical consultation, and an end goal of stabilization and disposition to the CT scanner for the CT head. Adjuncts were provided, if requested, including lab and imaging results (Appendix A). The case was completed in a traditional simulation format, running 10 minutes uninterrupted followed by 20 minutes of debriefing and discussion. We felt running the first case in a traditional format allowed the instructors to gauge the level of the learner group as well as to identify key teaching points to address in the subsequent RCDP rounds.

The second case, involving penetrating gunshot wounds, used RCDP with three rounds of increasing severity (Appendix B). For round 1, the mannequin was prepared with a bullet wound in the anterior left abdomen, round 2 had multiple abdominal bullet wounds, and round 3 had both chest and abdominal bullet wounds. Patient stability declined with each round, and the complexity of interventions required increased proportionally. Interventions required by the end of the final round included needle decompression with subsequent chest tube placement, intubation, intraosseous line placement, blood transfusion, activation of massive transfusion protocol, and emergent disposition to the operating room. We utilized a separate task trainer for chest tube placement. Lab and imaging results were provided if requested by the team (Appendix B).

During successive rounds for case 2, team members changed roles with each round to ensure all had the opportunity to act in each position appropriate for their role. Roles for physicians and APPs included team lead, airway/primary survey, secondary survey, and bedside procedures. For nursing staff and EMTs, roles included bedside nursing procedures, medication/blood administration, placement of bullet markers prior to imaging, and documentation.

We involved all instructors and learners in the debriefing session after the first case, as well as giving feedback and learning points during RCDP rounds of the second case. Instructors took 3–5 minutes after each case/round to reset the mannequin and equipment.

### Debriefing

We utilized a session instructor guide with critical action points for each case. For the traditional-style first case, we reviewed each of these points in the debriefing afterwards. In the second case progression, the instructors stopped and addressed each critical action in real time (Appendices A–C).

Our first case, which was in traditional simulation style, stopped after approximately 10 minutes, with the end goal of completing the primary survey and necessary interventions, selecting an appropriate disposition, and completing a full secondary survey (optional). We then moved to the debriefing room and utilized PEARLS,^18^ as discussed in Appendix C. The time goal for the first case plus debriefing session was 30 minutes in total.

For each round of case 2, we utilized RCDP to identify key pausing or stopping moments to address learning opportunities within the case in real time at the bedside (Appendices B and C). At the end of each round, a brief recap was conducted to discuss key points. Additionally, we developed key points entitled Modified ATLS Principles for Penetrating Trauma (Appendix D) specific to primary assessment of penetrating injuries; these principles were discussed during the debrief. The total time allotted for all three rounds of case 2 including debriefing and skills was 75 minutes.

### Assessment and Evaluation

Postsimulation evaluations gathered both quantitative and qualitative feedback. We asked the learners to rate effectiveness, clarity, quality of teaching, and the session overall, as well as to rate and provide feedback to the individual instructors. During the initial academic year 2017–2018, written surveys were distributed that utilized a single Likert scale for overall rating of the session, as well as free-text opportunities to provide qualitative feedback; the surveys were returned in an anonymous manner. For the subsequent four academic years, an electronic REDCap (research electronic data capture)^20^ form with 5-point Likert scales (1 = *Strongly Disagree,* 5 = *Strongly Agree*) was utilized to rate session quality through 10 different responses; individual instructor ratings and subjective feedback and comments were also solicited (Appendix E). All feedback was anonymous. All instructors participated in review of the cases, sessions, feedback, and survey results and in revising goals, objectives, and cases as deemed necessary at least once annually.

## Results

From 2017 to 2021, we conducted 25 sessions with 134 learners total. These learners included 33 surgery residents, five surgical fellows, 17 surgical APPs, 29 PEM fellows, two PEM faculty, three EC APPs, 32 EC nurses, 10 RTs, and three EC EMTs.

For the initial academic year and written survey, we received 23 out of 30 surveys, a response rate of 77%. The mean Likert score during this period was 4.7 out of 5.0 for overall session rating. Written feedback and responses are included in the overall qualitative results below.

From August 2018 through December 2021, postsession evaluations were collected via electronic surveys. We captured 76 surveys out of 104 learners during this period, a response rate of 73%. Quantitative feedback on the overall session quality was universally high (Table). The highest scoring categories included “I plan to apply what I learned here to my clinical practice,” with an overall mean of 4.8 out of 5.0, and “I would recommend this session to my colleagues,” with an overall mean of 4.7 out of 5.0. Ratings for “I feel that the session has improved my communication skills” and “I feel that the session has improved my ability to care for my patients” were also high, both with mean overall scores of 4.7 out of 5.0. Mean scores did not significantly vary from group to group when looking at physicians versus nurses or at surgeons versus PEM learners. The group scoring the session the highest was the three EMTs, with all ratings being 5.0 out of 5.0. The Table reflects mean scores overall, as well as scores divided by learner clinical role and department.

**Table.**
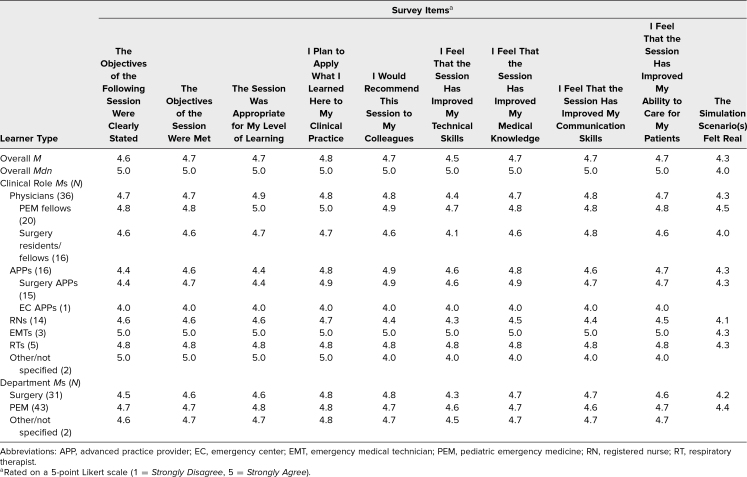
Postsession Survey Scores: Overall (*N* = 76) and by Learner Type

Qualitative feedback was overall very positive. The most frequent positive feedback included themes of practicing as a multidisciplinary team, the primary focus on team communication, and the opportunity to practice with members of the surgical and PEM teams together in a low-stress environment. Learners expressed preference for the RCDP format of the rounds in case 2, “talking through the simulation, pausing and restarting” to practice best case management. Learners from both surgery and PEM noted they enjoyed practicing the different roles, “learning about roles of the different team members,” and “rotating roles to give multiple perspectives.” Many reflected on the benefit of specific attention to managing penetrating injuries in contrast to blunt trauma. Opportunities for improvement included having more time to practice procedures, having ultrasound available for practice, and adding additional cases with more rare traumatic injuries, procedures, and/or management.

## Discussion

Timely and accurate trauma survey completion incorporating clear communication and teamwork amongst a trauma team is critical for ideal patient care and outcomes. This training session allows a multidisciplinary team to practice in a manner reflecting real-life team composition and experience levels. Additionally, it provides the opportunity to practice care for both blunt and penetrating injuries in pediatric trauma, as well as associated procedures and system processes.

To our knowledge, this is the first published pediatric trauma simulation training session utilizing both traditional and RCDP formats, as well as incorporating both blunt and penetrating injuries in a multidisciplinary format. We found that the multidisciplinary nature of both learners and instructors provided the broadest and most constructive teaching and was the most beneficial component of the session per the learners.

Our simulation sessions were implemented in August 2017 and continued with review and revisions through December 2021. The initial cases included a blunt trauma case in traditional format, followed by a penetrating trauma case in RCDP format involving the abdomen, chest, and neck. Scenarios were revised and adapted over the next five academic years based on feedback from instructors and learners. Changes to the first blunt trauma scenario included modifying the prearrival information, minimizing prompting to consider abuse as the presenting cause of trauma, and adding scenario specifics including vitals and clinical findings to lead the team through stabilization, with the goal of obtaining imaging prior to transfer to the operating room (OR). Changes to the second penetrating trauma scenario included altering locations of the gunshot wounds in each RCDP round. Initially, the patient had an abdominal wound, followed by abdominal and thoracic, and then thoracic and neck. Based on feedback from the surgeons involved, we finalized cases with progressive severity of abdominal and then abdominal and thoracic trauma to facilitate discussion from the surgical residents and fellows on triaging OR priorities, including a decision on which compartment or body cavity to explore first. The penetrating neck injury was removed from the final version.

After the first academic year of the session and based on qualitative feedback from learners, we amended the session to include clearer delineation of roles, teaching points specific to penetrating trauma (Appendix D), and task trainers for skills practice including chest tube placement. Also prompted by feedback, we invited additional participants to bolster the multidisciplinary team, including EC EMTs and RTs. When possible, we added a 15-minute trauma bay tour in the EC, led by a trauma nurse educator, for finding key trauma equipment. Implementation and further rounds of practice and development were limited by the COVID-19 pandemic and postpandemic resource utilization, namely, constraints on the personnel available to participate and instruct.

Although we felt the verbal and written feedback from learners and instructors throughout these sessions was robust and informative, the lack of quantitative data on achievement of educational objectives and on how these sessions impacted true clinical performance is a limitation of this project. For the future, we have discussed collecting pre- and postsession surveys to assess both medical knowledge and communication skills, as well as utilizing data from real patient encounters via video review to compare performance of individual learners before and after training sessions.

Moving forward, we are utilizing gathered feedback and combined instructor experiences to direct adaptation of multidisciplinary trauma simulation experiences. We are conducting in situ simulations in the EC and elsewhere, such as the pediatric intensive care units, as well as expanding learner groups to include pediatric and emergency medicine residents and PEM attending physicians.

## Appendices


Simulation Case 1.pdfSimulation Case 2.pdfDebriefing Materials.docxModified ATLS Principles for Penetrating Trauma.pdfEvaluation.pdf
*All appendices are peer reviewed as integral parts of the Original Publication.*
