# Supplementary material for: Multidisciplinary Simulation for Blunt and Penetrating Pediatric Trauma Utilizing Standard and Rapid Cycle Deliberate Practice Models
Source: MedEdPORTAL. 2024 Mar 19;20:11390. doi: 10.15766/mep_2374-8265.11390 (PMC10948622; doi:10.15766/mep_2374-8265.11390)
Supplement: Supplementary file 1 — Simulation Case 1.pdfSimulation Case 2.pdfDebriefing Materials.docxModified ATLS Principles for Penetrating Trauma.pdfEvaluation.pdf [file mep_2374-8265.11390-s001.zip › D. Modified ATLS Principles for Penetrating Trauma.pdf]

Modified ATLS® Principles for Penetrating Trauma
Gunshot Wound (GSW) Management

- **A** – Don't intubate unless absolutely necessary (defer to Operating Room)
- **B** – Chest GSW→ place chest tube on injured side
- **C** – Tourniquet/pressure/blood, activate Massive Transfusion Protocol
- **D** – Doesn't matter unless GSW to head or combined blunt trauma
- **E** – Roll and identify all bullet holes to assess injury locations
- Secondary survey – limit to bullet trajectory (based on Xrays)
- Disposition – Get to the Operating Room as fast as possible

Minutes Matter!
