# Supplementary material for: Multidisciplinary Simulation for Blunt and Penetrating Pediatric Trauma Utilizing Standard and Rapid Cycle Deliberate Practice Models
Source: MedEdPORTAL. 2024 Mar 19;20:11390. doi: 10.15766/mep_2374-8265.11390 (PMC10948622; doi:10.15766/mep_2374-8265.11390)
Supplement: Supplementary file 1 — Simulation Case 1.pdfSimulation Case 2.pdfDebriefing Materials.docxModified ATLS Principles for Penetrating Trauma.pdfEvaluation.pdf [file mep_2374-8265.11390-s001.zip › E. Evaluation.pdf]

# Trauma Simulation Evaluation

*Please complete the survey below. Thank you!*

**Clinical Role**

- ○ Physician
- ○ RN
- ○ APP
- ○ Respiratory Therapist
- ○ EMT
- ○ Other

**Your Department**

- ○ PEM
- ○ Surgery

| | Strongly Disagree | Disagree | Neutral | Agree | Strongly Agree |
|---|---|---|---|---|---|
| **The objectives of the following session were clearly stated.** | O | O | O | O | O |
| **The objectives of the session were met.** | O | O | O | O | O |
| **The session was appropriate for my level of learning.** | O | O | O | O | O |
| **I plan to apply what I learned here to my clinical practice.** | O | O | O | O | O |
| **I would recommend this session to my colleagues.** | O | O | O | O | O |
| **I feel that the session has improved my technical skills.** | O | O | O | O | O |
| **I feel that the session has improved my medical knowledge.** | O | O | O | O | O |
| **I feel that the session has improved my communication skills.** | O | O | O | O | O |
| **I feel that the session has improved my ability to care for my patients.** | O | O | O | O | O |
| **The simulation scenario(s) felt real.** | O | O | O | O | O |

**Please give general comments/feedback on the session.**

---

**What was the best part of this session?**

---

**What can we do to make the session better?**

---

*Please provide feedback on your Instructors.*

**Name of Instructor 1 ___________________________**

| | Strongly Disagree | Disagree | Neutral | Agree | Strongly Agree |
|---|---|---|---|---|---|
| **The instructor set the stage for an engaging learning experience.** | O | O | O | O | O |
| **The instructor maintained an engaging context for learning.** | O | O | O | O | O |
| **The instructor structured the debriefing in an organized way.** | O | O | O | O | O |
| **The instructor provoked in-depth discussions that led me to reflect on my performance.** | O | O | O | O | O |
| **Any additional feedback for Instructor 1?** | | | | | |

**Name of Instructor 2 ___________________________**

| | Strongly Disagree | Disagree | Neutral | Agree | Strongly Agree |
|---|---|---|---|---|---|
| **The instructor set the stage for an engaging learning experience.** | O | O | O | O | O |
| **The instructor maintained an engaging context for learning.** | O | O | O | O | O |
| **The instructor structured the debriefing in an organized way.** | O | O | O | O | O |
| **The instructor provoked in-depth discussions that led me to reflect on my performance.** | O | O | O | O | O |
| **Any additional feedback for Instructor 2?** | | | | | |

**Name of Instructor 3 ___________________________**

| | Strongly Disagree | Disagree | Neutral | Agree | Strongly Agree |
|---|---|---|---|---|---|
| **The instructor set the stage for an engaging learning experience.** | O | O | O | O | O |
| **The instructor maintained an engaging context for learning.** | O | O | O | O | O |
| **The instructor structured the debriefing in an organized way.** | O | O | O | O | O |
| **The instructor provoked in-depth discussions that led me to reflect on my performance.** | O | O | O | O | O |
| **Any additional feedback for Instructor 3?** | | | | | |
