# Supplementary material for: Multidisciplinary Simulation for Blunt and Penetrating Pediatric Trauma Utilizing Standard and Rapid Cycle Deliberate Practice Models
Source: MedEdPORTAL. 2024 Mar 19;20:11390. doi: 10.15766/mep_2374-8265.11390 (PMC10948622; doi:10.15766/mep_2374-8265.11390)
Supplement: Supplementary file 1 — Simulation Case 1.pdfSimulation Case 2.pdfDebriefing Materials.docxModified ATLS Principles for Penetrating Trauma.pdfEvaluation.pdf [file mep_2374-8265.11390-s001.zip › A. Simulation Case 1.pdf]

Appendix A: Simulation Case #1

SIMULATION CASE TITLE #1: Blunt Trauma Case

AUTHORS: Henkel, Lemke, Naik-Mathuria, Gautreaux, Doughty

LEARNER AUDIENCE: Surgery physician trainee (1 or 2 upper-level residents or fellows) or surgery advanced practice provider (APP) (2), Pediatric Emergency Medicine (PEM) physician (1-2 fellows or attendings), Emergency Center (EC) nurses (2) or Emergency Medical Technician (EMT) (2), EC Respiratory Therapist

PATIENT NAME: Charles

PATIENT AGE: 12 months old

CHIEF COMPLAINT: Intracranial Injury, intra-abdominal injury, seizure

PHYSICAL SETTING: Resuscitation Bay of Emergency Center

Blunt Trauma Case

**Brief narrative description of case**	*12-month-old child who has intracranial and intraabdominal injuries from undisclosed abusive trauma.*
**Primary Learning Objectives**	1. Identify and assign roles at pre-arrival and arrival<br>2. Demonstrate the coordination of receiving the patient and Emergency Medical Services (EMS) handoff<br>3. Perform primary survey in order, and within 5 minutes<br>4. Use crisis resource management skills (assume/ assign clear roles, use closed loop communication, share mental model)<br>5. Perform secondary survey<br>6. Consider inflicted physical abuse as diagnosis
**Critical Actions**	1. During preparation phase assign roles and prepare equipment<br>2. Activate trauma alert and neurosurgery call<br>3. Perform primary survey. Items that are often missed:<br>a. Maintain c-spine immobilization during airway assessment<br>b. Initiate fluid resuscitation (blood or crystalloid)<br>c. Use modified Glasgow Coma Score (GCS) for non-verbal child<br>d. Consider mannitol or hypertonic saline<br>e. Obtain vital signs including temperature<br>4. Express differential diagnosis including abusive blunt trauma with mismatch of injury pattern and history provided.<br>5. Perform secondary survey from head to extremities and back
**Learner Preparation or Prework**	*Ensure the psychological safety of the learners by reviewing expectations and orienting them to the simulation environment including the space and equipment. Review the objectives and agenda including that the first scenario will run around 10 minutes and be followed by a debriefing.*

INITIAL PRESENTATION			
**Initial vital signs**	Heart Rate (HR) 100, Blood Pressure (BP) 65/30, Respiratory Rate (RR) 18, Temperature (T) 97.8ºF, O_2 saturation (SpO2) 95% on Room Air (RA)		
**Room Setup**	Trauma Resuscitation Room<br>Equipment check list:<br>• Monitors: Electrocardiogram leads, pulse ox, BP cuff, thermometer<br>• Airway: Nonrebreather, self-inflating resuscitation bag, laryngoscope with blades, endotracheal tubes of various sizes, stylets<br>• Cervical collar (c-collar)<br>• Backboard<br>• Broselow tape<br>• Intravenous access supplies<br>• Intraosseous access supplies (manual or automatic)<br>• Focused Assessment with Sonography in Trauma (FAST) results<br>• Lab result cards<br>• Blood and blood warmer<br>• Iso-tonic crystalloid fluid bags<br>• IV tubing for fluids and blood products<br>• Pediatric Glasgow Coma Scale (GCS) card<br>• Trauma flowsheet (paper or electronic version)<br><br>Mannequin information:<br>• Infant mannequin with eyeshadow bruising to abdomen below diaper on a backboard with improvised c-collar in place. Keep infant under sheet during report and while preparation phase of the team is completed		
**Embedded Participants**	***Emergency Medical Systems (EMS) Member (pre-arrival report by phone):*** *12-month-old boy, former full term, healthy child, coming via ambulance. Reported seizure activity at home. No longer actively seizing, now post-ictal with GCS 13*<br>*Vitals in field: HR 100, BP 76/30, RR 18, T 97.8, SpO2 95% on RA*<br>*Expected arrival time is 2 minutes*<br><br>***EMS Member (arrival report):*** *12-month-old boy, previously healthy, fall from changing table at home with seizure lasting approximately 10 minutes*<br>*[Embedded participant playing EMS Member can leave and play part of surgery attending too]*<br><br>***Surgery Attending (at end of case):*** *Asks for and receives report.*		
**HPI**	*12-month-old boy, previously healthy, fall from changing table at home with seizure lasting approximately 10 minutes*		
**Past Medical/Surgical History**	**Medications**	**Allergies**	**Family/ Social History**

None	None	None known	Lives with mother and her boyfriend

**Physical Examination: (if not filled in, normal)**	
**Airway/ Breathing**	Patent, breathing 18 times a minute, clear lungs on auscultation, Sat 95%
**Circulation**	HR 100, BP 65/30, strong pulses, 2-3 second cap refill
**Disability/ CNS**	GCS 9: eyes open to pain (2), cries to pain (3), withdraws to pain (4)<br>(*provide each piece of GCS when asked)*
**Exposure**	Temp 97.8F, Bruising on lower abdomen (under diaper edge)

**INSTRUCTOR NOTES**		
**Expected Intervention / Time point**	**Change in Case**	**Additional Information**
Identify need for upgrade to Code 1 trauma based on vitals	If not identified, further drop blood pressure to 55/25	
Provide supplemental oxygen via non-rebreather	Improve saturations to 100%	If decline in mental status not noted, decrease RR to 10, sats to 89%
Give blood: plasma or pRBC 10 ml/kg	Lower HR by 5, Increase BP to 100/40	Patient in hemorrhagic and neurogenic shock, HR depressed by increased ICP
Identify traumatic brain injury, consider hypertonic saline or mannitol	No further decline in respiratory or mental status if given	
Identify abdominal bruising, concern for abuse, order trauma labs and consider CT abdomen/pelvis	If not identified, have embedded participant prompt removal of diaper revealing bruising	Provide lab results if appropriate indicating elevated transaminases
Stabilize for transport to CT	If not identified or suggested transport directly to OR, have surgery/neurosurgery team member request imaging	

Ideal Scenario Actions

- Pre-arrival: Team lead activates Code 2 trauma alert, leads pre-arrival team huddle and pages neurosurgery for suspicion of intracranial injury.

- Arrival: Upgrade to Code 1 trauma

- Airway/ Breathing:
    - Provide supplemental oxygen via non-rebreather.
    - Maintain c-spine stabilization, place C-collar.

- Consider and set up for intubation given declining mental status.

- Cardiovascular:
    - Identify hemorrhagic shock: Start with 10ml/kg of blood products, plasma if available, or pRBC. If no blood products, give 20 ml/kg isotonic fluid.
    - Consider focused ultrasound examination to look for intra-abdominal hemorrhage.

- Disability:
    - Recognize declining mental status GCS 13 → 9 (team should calculate), traumatic brain injury, order CT head.
    - Consider hypertonic saline and/or mannitol to treat increased intracranial pressure.

- Exposure:
    - Maintain temperature while performing head to toe examination.
    - Recognize abdominal bruising and concern for abusive/inflicted injuries. Order full trauma panel of labs, consider CT abdomen/pelvis

- Summary and hand-off
    - Team lead to provide mental model summarizing statement to lead surgeon and prepare team to transport to CT

Lab and Imaging Results to provide team when asked:
Hemoglobin 8, AST 356, ALT 492, VBG 7.38/45, lactate 3
Chest Xray normal
E-FAST results: +free fluids in the right upper quadrant and suprapubic space
Provide your own images/clips if possible.

Suggested Time Frame for the Session:

Case 1: Team simulation	10 minutes
Traditional debrief	20 minutes
Case 2: RCDP Rounds with incorporated debriefing of each round	75 minutes
Final wrap up and review of key points:	15 minutes
**Total session time**	**120 minutes**
