# Supplementary material for: Multidisciplinary Simulation for Blunt and Penetrating Pediatric Trauma Utilizing Standard and Rapid Cycle Deliberate Practice Models
Source: MedEdPORTAL. 2024 Mar 19;20:11390. doi: 10.15766/mep_2374-8265.11390 (PMC10948622; doi:10.15766/mep_2374-8265.11390)
Supplement: Supplementary file 1 — Simulation Case 1.pdfSimulation Case 2.pdfDebriefing Materials.docxModified ATLS Principles for Penetrating Trauma.pdfEvaluation.pdf [file mep_2374-8265.11390-s001.zip › B. Simulation Case 2.pdf]

Appendix B: Simulation Case #2

SIMULATION CASE TITLE #2: Penetrating Trauma Case - Rapid Cycle Deliberate Practice (RCDP) Sequence

AUTHORS: Henkel, Lemke, Naik-Mathuria, Gautreaux, Doughty

LEARNER AUDIENCE: Surgery physician trainee (1 or 2 upper-level residents or fellows) or surgery advanced practice provider (APP) (2), Pediatric Emergency Medicine (PEM) physician (1-2 fellows or attendings), Emergency Center (EC) nurses (2) or Emergency Medical Technician (EMT) (2), EC Respiratory Therapist

PATIENT NAME: Jimmy

PATIENT AGE: 18 months old

CHIEF COMPLAINT: Penetrating Trauma: abdomen and chest

PHYSICAL SETTING: Resuscitation Bay of Emergency Center

Case #2, Round 1: Penetrating injury with Airway assessment, Breathing assessment, CIRCULATION: Hypotensive Shock, Disability exam, EXPOSURE: find bullet wounds

**Brief narrative description of case**	*18-month-old toddler who was shot in the abdomen and is in hypovolemic hemorrhagic shock.*
**Primary Learning Objectives**	*1.* Identify and assign roles at pre-arrival and adjust on arrival<br>*2.* Demonstrate the coordination of receiving the patient, EMS handoff, and the physical exam<br>*3.* Perform primary survey in order, within 5 minutes<br>*4.* Use crisis resource management skills (assume/ assign clear roles, use closed loop communication, share mental model)<br>*5.* Determine priorities during advanced trauma life support and adjust accordingly to care for a patient with penetrating trauma
**Critical Actions**	*1.* Recruit resources by sending level I trauma alert page and requesting blood to be present at bedside<br>*2.* Assess airway and breathing<br>*3.* Assess circulation and administer blood products to stabilize hemorrhagic hypovolemic shock<br>*4.* Identify all wounds, obtain plain x-ray with use of radio-opaque markers highlighting bullet wounds to identify injury path(s)
**Learner Preparation or Prework**	Pre-Briefing: Explain RCDP format

Initial Presentation

**Initial vital signs**	Heart Rate (HR) 170, Blood Pressure (BP) 62/32 Respiratory Rate (RR) 22, Temperature (T) $97.8^{o}F$, O_2 saturation (SpO_2) 97% on non-rebreather		
**Room Setup**	Trauma Resuscitation Room<br>Equipment check list:<br>• Monitors: Electrocardiogram leads, pulse ox, BP cuff, thermometer<br>• Airway: Nonrebreather, self-inflating resuscitation bag, laryngoscope with blades, endotracheal tubes of various sizes, stylets<br>• Cervical collar (c-collar)<br>• Backboard<br>• Broselow tape<br>• Intravenous access supplies<br>• Intraosseous access supplies (manual or automatic)<br>• Focused Assessment with Sonography in Trauma (FAST) results (see below)<br>• Lab results (see below)<br>• Blood products (plasma, packed red blood cells) and blood warmer<br>• Iso-tonic crystalloid fluid bags<br>• IV tubing for fluids and blood products<br>• Pediatric Glasgow Coma Scale (GCS) card<br>• Radio-opaque markers to label wound locations on X-ray<br><br>Mannequin information:<br>• Toddler mannequin with moveable **bullet wounds** (plastic molded/drawn wounds with removable double-sided tape), first wound located on right anterior abdomen (with gauze covering) and second wound on posterior left abdomen. Patient is brought on a stretcher with a nonrebreather mask on and without a c-collar in place.		
**Embedded Participants**	***EMS Member (pre-arrival report by phone)*** *18-month-old toddler playing in front yard, fight broke out on street and guns fired, child has a bullet wound to the abdomen*<br>*Vitals: HR 170, BP 62/32, RR 22, SpO2 97% on nonrebreather*<br>*Expected arrival in 2 minutes*<br><br>***EMS Member (arrival report):*** *18-month-old toddler playing in front yard, fight broke out on street and guns fired, child has a bullet wound to the abdomen.*<br>*We were unable to establish access in the field, no medications given, placed on oxygen. [Embedded participant playing EMS member can leave and play part of surgery attending also.]*<br><br>***Surgery Attending (at end of case):*** *Asks for and receives report.*		
**HPI**	*18-month-old toddler playing in front yard, fight broke out on street and guns fired, child has a bullet wound to the abdomen*		
**Past Med/Surg History**	**Medications**	**Allergies**	**Family/ Social History**
None	None	None known	Lives with parents
**Physical Examination: (if not filled in, normal)**			

**Airway/ Breathing**	Patent, breathing 22 times a minute, clear to auscultation, SpO2 97% on non-rebreather	
**Circulation**	HR 170, BP 62/32, strong pulses, 1 second capillary refill	
**Disability/ CNS**	GCS 14: eyes open (4), crying spontaneously (4), moves normally (6) *(provide each piece of GCS when asked)*	
**Exposure**	Temp 97.0F, Bullet wound on anterior right abdomen and posterior left back	
**Instructor Notes - Changes**		
**Expected Intervention / Time point**	**Change in Case**	**Additional Information**
Give Blood	Lower HR to 145, Increase BP to 80/40	*Early Responder Physiology*

Circulation, Exposure, Mental Model

Basics: ABCDE Approach

Initiate Access/ Fluids or Blood

Early search for bullet pathway

Ideal Scenario

Pre-arrival

- Receive phone report
- HARD STOP — Activate Level I Trauma Code, request blood to bedside, assign roles and plan initial actions after arrival (see below)

Arrival of Patient

- Move patient to bed
- List of simultaneous actions:
    - *Bedside provider:* Apply monitors then obtain estimated weight via length-based dosing tape
    - Soft Stop — *Primary Survey Physician:* Assess Airway/ Breathing and Circulation (but do not report findings until asked)
    - *Bedside provider:* Remove clothes, establish IV
    - *Team Lead/ Recorder*: Request and listen to report from EMS.

Primary Survey

- Team lead asks for primary survey report
- HARD STOP — **Primary Survey Physician: "Airway is clear and midline, breath sounds are clear bilaterally, patient is tachycardic to 170, and hypotensive to 62/32 with one bullet hole noted on abdomen. Patient is awake and crying, GCS 14."**
- **Team Lead: "This is hypovolemic hemorrhagic shock, likely from intraabdominal hemorrhage, please initiate 10 ml/kg of plasma (or pRBC's), what is the weight?"**
    - Bedside provider: "Weight is 10 kg."
    - Soft Stop — Team Lead: Give 100 mL of plasma (or 100mL of packed red blood cells, or 200 mL of crystalloid if blood not available) and let's look for other gunshot wounds by looking head to toe and then rolling the patient, and as we find them cover with dressings and mark them with a radio-opaque marker please."
- Bedside providers start IV, give blood.
- Primary survey physician marks abdominal wound with marker.
- Team looks for wounds from head to toe, then rolls patient and finds second wound on back, calling out findings as examination progresses and wounds called back by recorder.
- ***As blood product given, HR and BP improve as noted above***
- HARD STOP — **Team Lead Mental Model**: **"To summarize, we have two bullet wounds, one on the right anterior abdomen and one on the left back. Let's obtain an X-ray now to see if there are any bullets or**

fragments in the abdomen or if this was a single entry and exit pair of wounds."

- E-FAST finds free fluid in peritoneum.
- Team lead reviews x-ray and notes no bullets.

HARD STOP

- Surgery Attending asks for summary, **team lead summarizes**: "18 month old with gunshot wounds to the abdomen presenting in hemorrhagic shock. Airway/Breathing normal, Circulation with hemorrhagic shock and an early responder to blood products, with GCS 14. This patient will need to go to operating room because of the bullet wound penetrating the peritoneum."
- Surgery Attending: "I confirm that we should get ready to move patient to the operating room."

Anticipated common errors:
Page out incorrect level of trauma activation
Not sending for blood pre-arrival
Delaying search/full exposure for additional bullet wounds or not using markers prior to x-ray
Incomplete communication/summarized mental modeling statements by team lead

Lab and Imaging Results to provide team when asked:
Hemoglobin 8, AST 200, ALT 250, lipase 200, lactate 4
E-FAST: free fluid in suprapubic space
Abdominal Xray: no metallic foreign bodies noted
Provide additional values as desired

Case #2, Round 2: Penetrating injury, adds DISABILITY altered mental status and AIRWAY emergent intubation management			
**Brief narrative description of case**	*18-month-old toddler who was shot in the abdomen and has hypovolemic hemorrhagic shock and altered mental status requiring airway protection*		
**Primary Learning Objectives (Additional to Round 1)**	1. Recognize depressed mental status and need for airway protection<br>2. List appropriate rapid sequence medications and equipment needed for intubation.		
**Critical Actions (Additional to Round 1)**	1. Gather appropriate airway supplies<br>2. Preoxygenate patient<br>3. Perform endotracheal intubation		
**Learner Preparation or Prework**	No additional work at this time		
**Initial Presentation**			
**Initial vital signs**	Heart Rate (HR) 180, Blood Pressure (BP) 62/32 Respiratory Rate (RR) 26, Temperature (T) 96.5°F, SpO_2 90% on non-rebreather, moaning during examination		
**Room Setup**	Changes from prior round:<br>Move gunshot wounds to new locations: right and left upper abdomen.		
**Embedded Participants**	***EMS Member (pre-arrival report):*** *18-month-old toddler playing in front yard, fight broke out on street and guns fired, child has a bullet wound to the abdomen.*<br>*Vitals: HR 180, BP 62/32, RR 26, SpO_2 90% on nonrebreather*<br>*Expected arrival in 2 minutes*<br><br>***EMS Member (arrival report):*** *18-month-old toddler playing in front yard, fight broke out on street and guns fired, child has bullet wounds to the abdomen.*<br>*We were unable to establish access in the field, no medications given, placed on oxygen.*<br>*[EMS Member can leave and return as surgery attending]*<br><br>***Surgery Attending (At end of case):*** *Asks for and receives report*		
**HPI**	*18-month-old toddler playing in front yard, fight broke out on street and guns fired, child has a bullet wound to the abdomen*		
**Past Medical/Surgical History**	**Medications**	**Allergies**	**Family/ Social History**
None	None	None Known	Lives with parents
**Examination: (If not filled in, normal)**			
**Airway/ Breathing**	Patent, breathing 26 times a minute, clear auscultation, SpO2 90% on non-rebreather		

**Circulation**	HR 180, BP 62/32, weak pulses, 3-4 sec capillary refill
**Disability**	GCS 8: Eyes open to pain (2), Moans to pain (2), Withdraws from painful stimuli (4)
**Exposure**	Temp 97, bullet wounds on anterior right and left upper abdomen.

Instructor Notes - Changes

**Expected Intervention / Time point**	**Change in Case**	**Additional Information**
Give Blood	Lower HR by 30 to 150, Increase BP to 70/40	*Early Responder Physiology*
Intubate/ effectively ventilate	Increase SpO2 to 98%	*Hypoxia was from disordered control of breathing.*

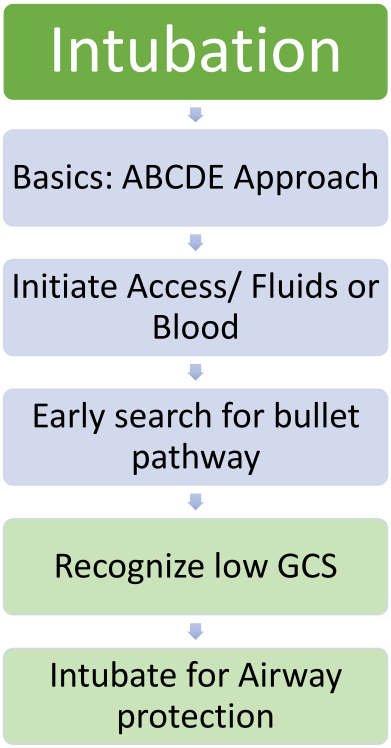

Ideal Scenario
Pre-arrival

- Receive phone report
- HARD STOP — Activate Level I Trauma Code, request blood to bedside, assign roles and plan initial actions after arrival (see below)

Arrival of Patient

- Move patient to bed
- List of simultaneous actions:
  - *Bedside provider:* Apply monitors then obtain estimated weight via length-based dosing tape
  - Soft Stop — *Primary Survey Physician:* Assess Airway/ Breathing and Circulation (but do not report findings until asked)
  - *Bedside provider:* Remove clothes, establish IV
  - *Team Lead/ Recorder*: Request and listen to report from EMS.

Primary Survey

- Team lead asks for primary survey report
- HARD STOP — **Primary Survey Physician "Airway is clear and midline, breath sounds are clear bilaterally, slow respirations and hypoxia noted so will begin ventilation with bag-valve-mask device, patient is tachycardic to 170, and hypotensive to 62/32 with one bullet hole noted on abdomen. Patient is awake and crying, GCS 8.**"
- Team Lead: "Continue ventilating the patient. This is hypovolemic hemorrhagic shock, likely from intraabdominal hemorrhage, please initiate 10 ml/kg of plasma (or packed red blood cells/pRBC's), what is the weight?"
- Bedside provider: "Weight is 10 kg."
- Team Lead: Give 100 mL of plasma (or 100mL of pRBC's, or 200 mL of crystalloid if blood not available) and let's look for other gunshot wounds by looking head to toe and then rolling the patient, and as we find them cover with dressings and mark them with a radio-opaque marker please. We will reassess mental status as we ventilate and give fluids, but this patient will likely require airway protection." *[This represents appropriate prioritization of circulation above intubation for a depressed mental status since correction of hypotension may improve mental status.]*
- Bedside providers continue ventilations, start IV, give blood.
- Primary survey physician marks abdominal wound with marker.
- Soft Stop — Team looks for wounds from head to toe, then rolls patient and finds second wound on back, calling out

findings as examination progresses and wounds called back by recorder.

- E-FAST finds free fluid in peritoneum.
- ***As blood given, HR and BP and SpO_2 improve as noted above***
- HARD STOP **Team Lead Mental Model**: "To summarize, we have two bullet wounds on right anterior abdomen and left back. Let's obtain an x-ray now to see if there are any bullets or fragments in the abdomen or if this was a single entry and exit pair of wounds."
- Team lead reviews x-ray and notes no bullets. Summarizes: Airway is currently patent, breathing well, we are working on circulation and patient will require going to operating room. Given the low GCS 8, we can intubate here or in the operating room."
- **Surgery Attending:** "Since operating room is still getting prepped, lets proceed with intubation prior to transport."
- Intubation takes place with etomidate and rocuronium for induction and paralysis. Endotracheal intubation with video assistance.

Anticipated common errors:
Intubation prior to stabilization of circulation. This leads to a delay in the search for bleeding sites and may delay transport to operating room where definitive care can take place.

Lab and Imaging Results to provide team when asked:
Hemoglobin 8, AST 200, ALT 250, lipase 200, lactate 4
E-FAST: free fluid in right upper quadrant and suprapubic space
Abdominal X-ray: no metallic foreign bodies noted
Chest X-ray (after intubation): endotracheal tube in adequate position, equal expansion of bilateral lungs
Provide additional values as desired

Case #2, Round 3: Penetrating injury, adds BREATHING pneumothorax and chest tube			
**Brief narrative description of case**	*18-month-old toddler who was shot in the chest and abdomen and has tension pneumothorax, hypovolemic hemorrhagic shock, and altered mental status requiring airway protection*		
**Primary Learning Objectives (Additional to Rounds 1+2)**	1. Recognize tension pneumothorax<br>2. Prioritize management of tension pneumothorax over circulation and altered mental status		
**Critical Actions (Additional to Rounds 1+2)**	1. Perform needle decompression<br>2. Perform chest tube placement		
**Learner Preparation or Prework**	No additional work at this time		
**Initial Presentation**			
**Initial vital signs**	Heart Rate (HR) 180, Blood Pressure (BP) 62/32 Respiratory Rate (RR) 12, Temperature (T) 96.5°F, SpO_2 90% on non-rebreather, moaning during examination		
**Room Setup**	Changes from prior round:<br>Move gunshot wounds to new locations: right anterior chest and right anterior abdomen<br><br>If unable to perform needle decompression and chest tube placement on primary mannequin, have additional task trainer, such as TraumaChild, in order to perform these skills. Cover with a sheet until ready to use.		
**Embedded Participants**	***EMS Member (pre-arrival report):*** *18-month-old toddler playing in front yard, fight broke out on street and guns fired, child has bullet wounds to abdomen and chest.*<br>*Vitals: HR 170, BP 62/32, RR 12,* SpO_2 *90% on nonrebreather*<br>*Expected arrival in 2 minutes*<br><br>***EMS Member (arrival report):*** *18-month-old toddler playing in front yard, fight broke out on street and guns fired, child has bullet wounds to the abdomen and chest.*<br>*We were unable to establish access in the field, no medications given, placed on oxygen.*<br>*[EMS Member can leave and return as surgery attending]*<br><br>***Surgery Attending (at end of case):*** *Asks for and receives report*		
**HPI**	*18-month-old toddler playing in front yard, fight broke out on street and guns fired, child has a bullet wound to abdomen and chest wall*		
**Past Medical/Surgical History**	**Medications**	**Allergies**	**Family/ Social History**

None	None	None Known	Lives with parents

**Examination: (If not filled in, normal)**	
**Airway/ Breathing**	Patent, breathing 12 times a minute, decreased breath sounds on right chest, SpO2 90% on non-rebreather
**Circulation**	HR 180, BP 62/32, weak pulses, 3-4 sec capillary refill.
**Disability**	Eyes do not open, no movement, no verbalization (GCS 3)
**Exposure**	Temp 97, bullet wounds on anterior right chest and right upper abdomen.

Instructor Notes - Changes

**Expected Intervention / Time point**	**Change in Case**	**Additional Information**
Needle decompression/ chest tube placement	Improve SpO_2 to 93%, HR reduces to 160; BP improves to 70/40, show that 200 mL of blood collected in collection apparatus	*Relief of obstructive shock state*
Give Blood	Lower HR by 15 to 145, Increase BP to 70/40	*Non-responder physiology*
Intubate/ effectively ventilate	Increase SpO_2 to 96%	*Reduced ventilation caused low oxygen*

Ideal Scenario

Pre-arrival

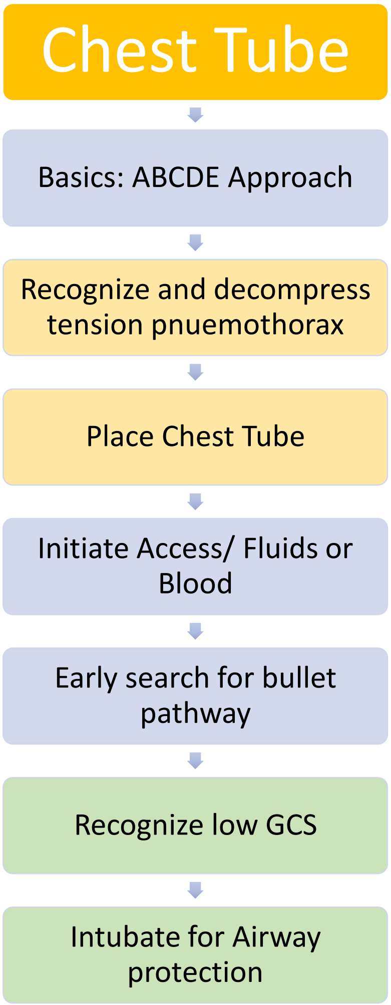

- Receive phone report
- HARD STOP — Activate Level I Trauma Code, request blood to bedside, request chest tube, assign roles and plan initial actions after arrival (see below)

Arrival of Patient

- Move patient to bed
- List of simultaneous actions:
    - *Bedside provider:* Apply monitors then obtain estimated weight via length-based dosing tape
    - Soft Stop — *Primary Survey Physician:* Assess Airway/ Breathing and Circulation (but do not report findings until asked)
    - *Bedside provider:* Remove clothes, establish IV
    - *Team Lead/ Recorder*: Request and listen to report from EMS.

Primary Survey

- Team lead asks for primary survey report
- HARD STOP — **Primary Survey Physician: "Airway is clear, shifted to left, breath sounds are decreased on right, slow respirations and hypoxia noted so will begin ventilation with bag-valve-mask device, patient is tachycardic to 170, and hypotensive to 62/32 with one bullet hole noted on abdomen. Patient is awake and crying, GCS 8."**
- Team Lead: "Continue ventilating the patient. Please needle decompress right chest with large-bore IV catheter. This is obstructive shock."
- Procedural Physician performs needle decompression and follows with chest tube placement.
- Team Lead: "As we relieve obstruction, this patient likely has hypovolemic hemorrhagic shock, from intraabdominal hemorrhage, please initiate 10 ml/kg of plasma (or pRBC's), what is the weight?"
- Bedside provider: "Weight is 10 kg."
- Team Lead: Give 100 mL of plasma (or 100mL of pRBC's, or 200 mL of crystalloid if blood not available) and let's look for other gunshot wounds by looking head to toe and then rolling the patient, and as we find them cover with dressings and mark them with a radio-opaque marker please. We will reassess mental status as we ventilate and give fluids, but this patient will likely require airway protection."
  [This represents appropriate prioritization of circulation above intubation for a depressed mental status, since correction of hypotension may improve mental status.]
- Bedside providers continue ventilations, start IV, give blood.
- Primary survey physician marks abdominal wound with marker.
- Soft Stop — Team looks for wounds from head to toe, then rolls patient and finds second wound on back, calling out findings as examination progresses and wounds called back by recorder.
- E-FAST finds free fluid in peritoneum.
- ***As blood given, HR and BP and SpO_2 improve as noted above***
- HARD STOP — **Team Lead Mental Model**: "To summarize, we have two bullet wounds on right anterior abdomen and right chest. We have a right sided chest tube placed with 200mL of output. We have given 1x 10ml/kg bolus of plasma and have improved perfusion. The next step will be to secure the airway with intubation, unless the operating room is ready."
- **Surgery Attending:** "Since the operating room is getting prepped, lets proceed with intubation prior to transport."
- Intubation takes place with etomidate and rocuronium for induction and paralysis. Endotracheal intubation with video assistance. Subsequent x-ray performed to check tube placement as well as evaluate for bullets in the chest and abdomen.
- Team lead prepares team for transport to the operating room

Anticipated common errors:
Intubation prior to recognition and treatment of tension pneumothorax.
Chest tube placement/ needle decompression can be difficult and require additional training.

Lab and Imaging Results to provide team when asked:
pH 7.12, pCO2 65, pO2 50, bicarb 11, lactate 6
Hemoglobin 6, AST 200, ALT 250, lipase 200
E-FAST: +hemothorax on right, +Free fluid in right upper quadrant, no fluid in pericardial sac
Include your own images/clips if possible
Chest Xray
If asked prior to chest tube, intubation: hemopneumothorax on the right
If asked after placement of tubes: chest tube and ETT in proper position with re=expansion of the lung
Abdominal Xray: bullet in the right upper quadrant with free air
Provide your own images if possible, and additional lab values as desired

Suggested Time Frame for the Session:

Case 1: Team simulation	10 minutes
Traditional debrief	20 minutes
Case 2: RCDP Rounds with incorporated debriefing of each round	75 minutes
Final wrap up and review of key points:	15 minutes
**Total session time**	**120 minutes**
