# Supplementary material for: Multidisciplinary Simulation for Blunt and Penetrating Pediatric Trauma Utilizing Standard and Rapid Cycle Deliberate Practice Models
Source: MedEdPORTAL. 2024 Mar 19;20:11390. doi: 10.15766/mep_2374-8265.11390 (PMC10948622; doi:10.15766/mep_2374-8265.11390)
Supplement: Supplementary file 1 — Simulation Case 1.pdfSimulation Case 2.pdfDebriefing Materials.docxModified ATLS Principles for Penetrating Trauma.pdfEvaluation.pdf [file mep_2374-8265.11390-s001.zip › C. Debriefing Materials.docx]

**Appendix C: Debriefing Materials**

Case #1, Traditional Simulation Format

After completion of the case, instructors and learners gathered in a separate debriefing space (outside the simulation room) to discuss the case and debrief. Instructors utilized PEARLS^15^ debriefing format:

Reaction: “How are each of you feeling after completing that case?”

*Elicit a word or a few words from each participant.*

Description: “Can someone summarize the case and the issues you addressed?”

*Have a team member summarize the medical aspects and performance.*

*An instructor can add in details or summarize if there are no volunteers.*

Analysis: “What aspects of the case went well and why?”

*Focus on positive actions and communication first.*

“What aspects would you do differently next time and why?”

*Address areas for opportunity in a broadly applicable manner.*

Summary: “What key point can you take away to apply in the future?”

*Elicit one point from each team member.*

Instructor/debriefer useful phrases:

“I noticed you did [x], next time you may try [y] because [z].”

“I liked that you said/did [a], what made you say/do that?”

**Key teaching points for Case #1**

Medical knowledge

- Complete primary survey per ATLS guidelines, goal within 5 minutes.
- Identify unstable patient and need to upgrade to Code 1 or highest-level trauma activation.
- Recognize shock, give blood products for presumed hemorrhagic shock in trauma.
- Recognize declining mental status with GCS 13 on scene, GCS 9 at arrival, need for emergent neurosurgical consult and CT head. Consider mannitol or hypertonic saline. Consider intubation for airway protection if further decline.
- Identify abusive/inflicted injuries concerning for child abuse.
- Perform secondary survey per ATLS guidelines after stabilization of primary.

Team communication and function

- Pre-arrival activation based on information available, review your facility’s trauma level activation criteria for Code 1 versus 2. Review criteria/indications for upgrading to highest level activation.
- Assign roles pre-arrival: team lead, airway/primary survey, secondary survey, procedures, medications, documentation.
- Mental model of clinical summary, interventions, and next steps after each intervention.
- Summarize disposition and hand-off to trauma surgery team.
- Closed-loop communication between team members and team lead.

Case #2, Rounds 1, 2 and 3 in Rapid-Cycle Deliberate Practice^9^ Style

This case format included pauses by the instructors during the case when key teaching points were identified. Similar phrasing was used: “Let’s pause – I noticed you said/did [x], I’d like you to try [y] because [z].” “Hard stops” as indicated in Appendix B were considered the most important points to pause if not executed optimally. “Soft stops” indicated additional break points to ensure key points were made. After the debriefing feedback was delivered, the instructor determined where to “rewind” the case to practice the same step again. The instructor could also choose to stop and restart the scenario from the beginning. At the beginning of each round, the learners changed roles so that all had a chance to participate as different members of the team, as appropriate for their role/training.

**Key teaching points for Case #2, Round 1**

Medical knowledge

- Complete primary survey per ATLS guidelines, goal within 5 minutes.
- Identify hemorrhagic shock and start blood products, ideally 10 ml/kg of plasma for volume expansion, or pRBC 10 ml/kg if plasma not available. Continue 1:1:1 transfusion of products as indicated.
- Utilize log roll as part of Exposure in penetrating trauma to identify all wounds.
- Utilize bullet markers and x-ray adjuncts to identify the location and trajectory of penetrating wounds/foreign bodies.
- Perform secondary survey per ATLS guidelines after stabilization of primary.

Team communication and function

- Pre-arrival: Activate Code 1 trauma, assign roles, prepare equipment.
- Mental model of clinical summary, interventions, and next steps after each intervention.
- Summarize disposition and hand-off to trauma surgery team.
- Closed-loop communication between team members and team lead.

**Key teaching points for Case #2, Round 2**

Medical knowledge

- Complete primary survey per ATLS guidelines, goal within 5 minutes.
- Identify hemorrhagic shock and start blood products, ideally 10 ml/kg of plasma for volume expansion, or pRBC 10 ml/kg if plasma not available. Continue 1:1:1 transfusion of products as indicated.
- Identify low GCS and need for intubation for airway protection. Utilize etomidate for sedation in trauma for maintaining normotension.
- Utilize log roll as part of Exposure in penetrating trauma to identify all wounds.
- Utilize bullet markers and x-ray adjuncts to identify the location and trajectory of penetrating wounds/foreign bodies.
- Perform secondary survey per ATLS guidelines after stabilization of primary.

Team communication and function

- Pre-arrival: Activate Code 1 trauma, assign roles, prepare equipment.
- Mental model of clinical summary, interventions, and next steps after each intervention.
- Summarize disposition and hand-off to trauma surgery team.
- Closed-loop communication between team members and team lead.

**Key teaching points for Case #2, Round 3**

Medical knowledge

- Complete primary survey per ATLS guidelines, goal within 5 minutes.
- Identify hemopneumothorax, perform needle decompression and chest tube placement.
- Identify hemorrhagic shock and start blood products, ideally 10 ml/kg of plasma for volume expansion, or pRBC 10 ml/kg if plasma not available. Continue 1:1:1 transfusion of products as indicated.
- Identify low GCS and need for intubation for airway protection.
- Utilize log roll as part of Exposure in penetrating trauma to identify all wounds.
- Utilize bullet markers and x-ray adjuncts to identify the location and trajectory of penetrating wounds/foreign bodies.
- Perform secondary survey per ATLS guidelines after stabilization of primary.

Team communication and function

- Pre-arrival: Activate Code 1 trauma, assign roles, prepare equipment.
- Mental model of clinical summary, interventions, and next steps after each intervention.
- Summarize disposition and hand-off to trauma surgery team.
- Closed-loop communication between team members and team lead.
